# Emergency Surgery in Urology during the COVID-19 Pandemic

**DOI:** 10.1590/S1677-5538.IBJU.2020.S125

**Published:** 2020-07-27

**Authors:** Marcelo Torrico de la Reza, Ana María Autrán-Gómez, Germán Urenda Tardío, Javier Arancibia Bolaños, Juan Carlos Gil Rivero

**Affiliations:** 1 “Los Olivos” Clinic Urology Service Cochabamba Bolivia “Los Olivos” Clinic Urology Service, Cochabamba, Bolivia; 2 Universidad Mayor de San Simón Cochabamba Bolivia Universidad Mayor de San Simón, Cochabamba, Bolivia; 3 University Hospital Fundación Jimenez Diaz Department of Urology Madrid Spain Department of Urology, University Hospital Fundación Jimenez Diaz, Madrid, Spain

**Keywords:** Urology, Emergencies, COVID-19 diagnostic testing [Supplementary Concept]

## Abstract

**Proposal:**

To highlight the indications for emergency surgery during the 2019 Coronavirus pandemic (COVID-19) that support recommendations published in midMarch 2020 by the American Confederation of Urology on its website.

**Materials and Methods:**

A bibliographic search was conducted in PubMed and Cochrane Library to perform a non-systematic review, using key words: Urology, Emergency and COVID-19, to determine recommendations for patients that should receive emergency care due to urological pathology.

**Results:**

The main recommendations and protocols in the management of different urological emergencies during the COVID-19 pandemic are reviewed and discussed.

**Conclusions:**

We are living a new condition with the COVID-19 pandemic, which obliges urologists to conform to the guidelines that appear on a daily basis formulated by multidisciplinary surgical groups to manage urological emergencies. Consequently, in this time of health crisis, we must adapt to the resources available, implementing all biosecurity measures to protect patients and all health personnel who are in charge of patient management.

## INTRODUCTION

The World has changed since the beginning of December 2019, when the city of Wuhan, China, identified the first case of an atypical pneumonia produced by a Coronavirus, which is now known as COVID-19. From that first case to date, the disease has had exponential growth, and the World Health Organization has classified it as a true pandemic (2). Up to May 15, 2020 (3), this disease has spread across more than 185 countries, affected more than 4,400,000 people, and killed more than 300,000 patients worldwide. The southern hemisphere is currently entering the winter season and is seeing an overwhelming upsurge of reported cases in this part of the planet.

This avalanche of cases has saturated the health systems of most countries, where a large number of patients have been reported, which has forced changes in the dynamics of hospitals. We are currently seeking solutions to optimize resources, restructuring spaces so that we can absorb patients that we potentially know have a great chance of transmitting the disease to other patients and to all the health personnel who are providing care; this may be aggravated in some places by the restriction or lack of personal protective equipment (4).

Most emergency services are full of patients, many of them with great need to be admitted to these hospitals. For this reason, operating structures of hospitals have been transformed and rooms have been filled with COVID 19 patients.

The need for beds and mechanical ventilators in intensive care units has increased due to the influx of critical patients requiring ventilatory support, transforming surgical areas into intensive care spaces, decreasing the capacity of surgical areas. On the other hand, it has been shown that patients and families who are coming to these hospitals with COVID 19 patients have increased the risks of being infected with this pandemic.

The large influx of patients in many places has forced urologists to actively participate in emergency services to support the care of COVID patients 19.

This has resulted in the need to postpone the care of pathologies of urology patients considered non-urgent. Scientific societies and institutions have made a number of recommendations that should be addressed as a matter of priority.

Urologists as well as all health personnel are forced to redefine our practice in consultation and emergency services, leading to the search for patient care protocols.

The intention of this review is to standardize which pathologies need emergency surgical care during this Covid 19 pandemic.

## OBJECTIVE

To highlight the indications for emergency surgery during the COVID 19 pandemic that support recommendations published in mid-March 2020 by the American Confederation of Urology (Table-1).

**Table 1:** American Urology Confederation (CAU) Position in the Surgical Management of Urologic Cases in COVID-19 Pandemic.

American Urology Confederation's position in the surgical management of urological cases during COVID-19 pandemic	
**First Recommendation**	Social Distancing Hand washing Fragile Patient Protection
**Genuine Emergency**	Renal colic, renal collections or abscesses, acute urinary retention, Fournier gangrene, Penis or testicle fracture, testicular torsion
**Absolute Oncologic Emergency**	Cystectomy, nephrectomy, TUR (high risk), high risk prostatectomy, orchiectomy, nephroureterectomy
**Non-oncologic Emergency**	Emergency lithiasis, hematuria with clot retention,
**Relative Oncologic Emergency**	Prostate biopsies, intermediate or low risk prostatectomy.
**Non-emergencies**	Scrotal surgery, cystoscopy, andrology surgery, brachytherapy, functional and reconstructive surgery, lithiasis elective surgery, HPB surgery.

## MATERIALS AND METHODS

A bibliographic search was conducted in PubMed and Cochrane Library to perform a non-systematic review, using key words: Urology, Emergency and COVID 19, to determine recommendations for patients that should receive emergency care due to urological pathology.

## RESULTS

Taking into account lessons learned from China, Italy, and other countries, in the coming weeks, rates of COVID-19 in the southern hemisphere are expected to start soaring and peaking at the end of May and June; there will be variability in rates, peaks, and times, and though we cannot predict what will happen at this point, we should all be preparing.

Accordingly, we are still recommending surgeons to reduce elective surgical procedures (5).

The American College of Surgeons has published a series of triage recommendations that guide the selection of non-emergency surgical procedures in COVID-19 patients (6) (Table-2).

**Table 2:** Elective Surgery Acuity Scale (ESAS).

Tiers/Description	Definition	Locations	Examples	Action
Tier la	**Low acuity surgery/ healthy patient** Outpatient surgery Not life threatening illness	HOPD ASC Hospital with low/no COVID-19 census	Carpal tunnel release Penile prosthesis EGD Colonoscopy	Postpone surgery or perform at ASC
Tier 1b	**Lowacuity surgery/ unhealthy patient**	HOPD ASC Hospital with low/no COVID-19 census		Postpone surgery or perform at ASC
Tier 2a	**Intermediate acuity surgery/healthy patient** Not life threatening but potential for future morbidity and mortality. Requires in hospital stay	HOPD ASC Hospital with low/no COVID-19 census	Low risk cancer Non urgent spine Ureteral colic	Postpone surgery if possible or consider ASC
Tier 2b	**intermediate acuity surgery/unhealthy patient**	HOPD ASC Hospital with low/no COVID-19 census		Postpone surgery if possible or consider ASC
Tier 3a	**High acuity surgery/ healthy patient**	Hospital	Most cancers Highly symptomatic patients	Do not postpone
Tier 3b	**High acuity surgery/ unhealthy patient**	Hospital		Do not postpone

### Guidelines for triage of cancer patients

Once the COVID 19 pandemic is confirmed, Hospital Centers and health personnel in charge of treating cancer patients should establish criteria for treating these patients based on criteria that have been defined by the American College of Surgeons. These criteria should be in relation to the availability of resources of the different centers: hospital and ICU beds, respirators, blood transfusion capacity and personal protective equipment, for patients and for all health personnel in charge of patients with this condition.

It is advisable so that only high-risk patients should be treated, advising hospitals to discontinue elective surgeries (6).

### Oncological pathology

Oncology patients have to visit hospitals because they have to receive treatment or be monitored for their disease, and they may be immunocompromised due to their malignant neoplasm or their cancer treatment, which puts them at risk of contracting infections and seems to have a higher risk of contracting COVID 19 than the general population.

Patients should be guided regarding hand hygiene measures, social distancing, use of personal protective equipment, and should be taught about symptoms and signs of the disease.

The need to carry out or postpone an active intervention must be individualized in low-risk patients and must be considered on a case-by-case basis.

Visits to hospital centers should be minimized and telemedicine programs should be incorporated to reduce the exposure risk (7).

In a study of 1,524 patients admitted to the Department of Radiation and Medical Oncology, in the Zhongnan Hospital of Wuhan University, from December 30, 2019 to February 17, 2020, it was revealed that cancer patients had a double risk of infection by COVID 19 compared to the general population (8).

The American Confederation of Urology has published on its website recommendations that oncological surgeries should be considered Genuine Emergencies: Cystectomy, Nephrectomy, Nephroureterectomy, Prostatectomy and TURB, all high risk and Orchiectomy for testicular tumor (Table-1).

The following were also considered as relative Oncological Emergencies: Prostate Biopsies, Prostatectomies of intermediate or low risk.

### Management of Lithiasis in the COVID 19 period

Management of renal colic should be done conservatively to the extent possible.

Patients who have a proven diagnosis of COVID 19 or who are highly suspicious and who need to undergo an endourology procedure should be managed in dedicated operating rooms, in negative pressure environments, ideally with regional anesthesia and if they need anesthesia machines, these should be used only for COVID 19 cases (9).

Despite the fact that lithiasis disease is considered a benign disease, many patients may present cases of renal colic resistant to medical treatment and many may be complicated by severe septic conditions, which may require emergency surgery (10).

We can consider urgent situations in patients, who have the following conditions, related to lithiasis disease, that require emergency surgery: solitary kidney, acute kidney failure, bilateral obstruction, colic that is resistant to medical treatment, and kidney stones or infected ureteral.

We must not forget that patients, despite having decompressed the urinary tract, administered antibiotics and other support measures, 15% will need to enter intensive care units, places today where the beds are full of patients with COVID 19 and that despite all the care, mortality will range between 8-10% (10).

### Other emergency conditions

There are several urologic emergency conditions that we will describe one by one.

**Acute Urinary Retention:** If possible, the urethra should simply be catheterized or, in its absence, a retropubic cystostomy should be performed. At this time of the COVID 19 pandemic, prostate surgery for benign pathology should be considered a non-emergency. In the case of patients with severe hematuria, this will be discussed in the section related to this condition (11).

**Severe Hematuria:** In the event that the patient presents severe hematuria, the cause must be identified, possible coagulation disorders should be studied, a urethral catheter should be placed and the bladder should be irrigated. If nevertheless hematuria persists, an endoscopic examination should be performed, fulguration of active sites of bleeding, and eventually perform endoscopic resections of bladder or prostate tumors, trying to minimize hospital stay.

**Genitourinary Trauma:** Patients suffering from trauma to the genitourinary tract should be evaluated and classified; assessing hemodynamic stability and severity of hematuria; stable renal trauma should be managed conservatively and if stable should be done on an outpatient basis; patients with severe bleeding or leaks should be managed with endovascular procedures and / or ureteral catheters. Hemodynamieally unstable patients, with penetrating, V-degree injuries, pulsating or expanding hematomas, should be explored (12).

Fractures of the penis and testicles should be explored immediately with closure of the tunica albuginea in the first case, and trying to preserve as much viable tissue as possible, in the second case. These patients should be managed on an outpatient basis (12).

**Acute Scrotum:** It must be surgically intervened immediately, untwisting the affected testicle and, if this is feasible, bilateral orchiopexy should be performed, failing that, orchiectomy of the affected gonad. These must also be outpatient procedures (13).

**Scrotal masses:** They must be drained on an outpatient basis and healing must be performed at home, with the idea that they be resolved by second intention.

**Fournier Gangrene:** It is a true urology emergency that must be resolved by extensive debridement within 24 hours of its presentation and by administering broad-spectrum antibiotics, preferably performing procedures with spinal blocks and home cures (14).

## CONCLUSIONS

We are experiencing a new condition with the COVID 19 pandemic, which forces us urologists to comply with the guidelines that appear daily formulated by multidisciplinary surgical groups, to manage urological emergencies, and we must adapt to the available resources, implementing all the measures of biosecurity to protect patients and all health personnel who are in charge of patient management, in these times of health crisis.
